# Exploiting the Molecular Basis of Oesophageal Cancer for Targeted Therapies and Biomarkers for Drug Response: Guiding Clinical Decision-Making

**DOI:** 10.3390/biomedicines10102359

**Published:** 2022-09-22

**Authors:** Sikhumbuzo Mbatha, Rodney Hull, Zodwa Dlamini

**Affiliations:** 1 SAMRC Precision Oncology Research Unit (PORU), SARChI Chair in Precision Oncology and Cancer Prevention (POCP), Pan African Cancer Research Institute (PACRI), University of Pretoria, Hatfield 0028, South Africa; 2 Department of Surgery, Faculty of Health Sciences, Steve Biko Academic Hospital, University of Pretoria, Hatfield 0028, South Africa

**Keywords:** tumor mutational burden, HPV, HIV, miRNA, alternative splicing, oesophageal cancer, miRNA, biomarkers

## Abstract

Worldwide, oesophageal cancer is the sixth leading cause of deaths related to cancer and represents a major health concern. Sub-Saharan Africa is one of the regions of the world with the highest incidence and mortality rates for oesophageal cancer and most of the cases of oesophageal cancer in this region are oesophageal squamous cell carcinoma (OSCC). The development and progression of OSCC is characterized by genomic changes which can be utilized as diagnostic or prognostic markers. These include changes in the expression of various genes involved in signaling pathways that regulate pathways that regulate processes that are related to the hallmarks of cancer, changes in the tumor mutational burden, changes in alternate splicing and changes in the expression of non-coding RNAs such as miRNA. These genomic changes give rise to characteristic profiles of altered proteins, transcriptomes, spliceosomes and genomes which can be used in clinical applications to monitor specific disease related parameters. Some of these profiles are characteristic of more aggressive forms of cancer or are indicative of treatment resistance or tumors that will be difficult to treat or require more specialized specific treatments. In Sub-Saharan region of Africa there is a high incidence of viral infections such as HPV and HIV, which are both risk factors for OSCC. The genomic changes that occur due to these infections can serve as diagnostic markers for OSCC related to viral infection. Clinically this is an important distinction as it influences treatment as well as disease progression and treatment monitoring practices. This underlines the importance of the characterization of the molecular landscape of OSCC in order to provide the best treatment, care, diagnosis and screening options for the management of OSCC.

## 1. Introduction

In most patients, oesophageal cancer is characterized by late presentation, resulting in poor outcomes. Patients tend to only consult their healthcare practitioners at these later stages due to a variety of factors. These include no noticeable symptoms during the early stages of the disease and a lack of biomarkers for early detection (Reviewed in [1]). Due to this lack of biomarkers, diagnosis currently relies on well-established methods of histology followed by staging imaging prior to planning any treatment (Reviewed in [2]). A further group of complications that is especially prevalent in developing countries arises due to challenges within the health care system structures. These can include poor referral patterns and existing inequalities within the healthcare system. Several studies have tested different proteins and genetic markers for their potential use as biomarkers to improve current methods for the diagnosis of oesophageal carcinoma [3,4]. However, the advances in sequencing, along with increasing numbers of genome-wide association studies (GWAS) and the application of this genomics data acquisition methods to cancer, has started to change this; this may potentially lead to early detection and the promise of precision oncology. Not only do these studies have the potential to uncover biomarkers that may potentially be useful in the early diagnosis and treatment of oesophageal cancer, but they can be used to characterize populations that have previously been neglected in genomic studies, such as Africans. Next-generation sequencing (NGS) of tumor-derived DNA and RNA can reveal multiple cancer-specific changes to the genome and transcriptome. Sequence mutations, insertions, deletions, rearrangements, copy number variations (CNVs), and loss of heterozygosity can all be revealed by sequencing DNA isolated from cancer cells. Sequencing of the transcriptome of cancer cells can reveal the presence of, gene fusions, alternately splice mRNA transcripts and changes in the transcript levels of mRNAs (coding genes) or small non-coding RNAs. This would give information concerning gene or non-coding RNA transcription profiles which would be specific to a particular type or stage of cancer [5].

The use of NGS in large-scale cancer genomics discovery projects has resulted in the elucidation of the underlying molecular basis and mechanisms for cancer development and progression in a variety of tissues, including the genetic drivers of cancer [5]. These cancer-specific molecular changes, whether they are gnomic, transcriptomic or epigenomic, may serve as useful biomarkers. This can only happen once they have been identified as being associated with diseased tissues and not to be present in in normal tissue [6]. These changes, therefore, hold the promise of serving as new diagnostic and prognostic tools and may complement or replace histological analysis in this regard [3].

## 2. Altered Gene Expression in OSCC

**Figure 1:**
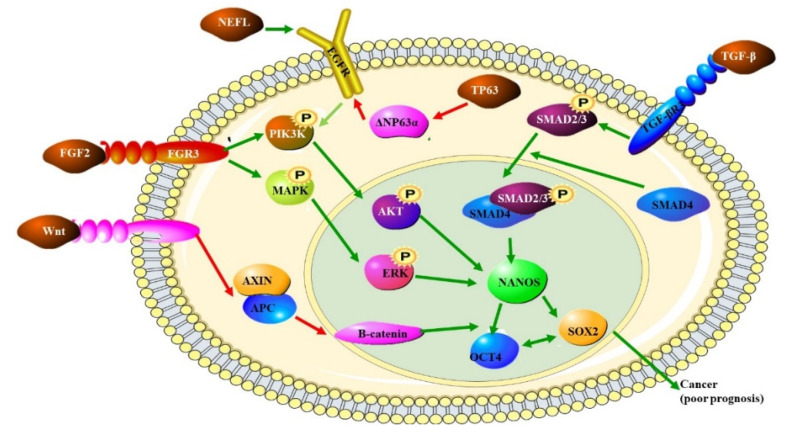
The role of various pathways in OSCC: the transcription factor SOX2, the ligand for the FRIZZLED receptor, WNT and the transcriptional activator TP63 are all up regulated in OSCC. The increased expression of these proteins also indicates a poor prognosis. SOX2 forms a trimeric complex with OCT4 on DNA and NANOS which controls the expression of several genes involved in proliferation. NANOS is a translational repressor which leads to the increased expression of metalloproteinases. Increased WNT expression leads to increased levels of β-catenin. TP63 inhibits the production of ΔNP63α, which dampens PIK3K by preventing EGFR signaling [10].

Oesophageal cancer develops from stratified squamous epithelium and is associated with molecular abnormalities in a variety of genes. These include structural chromosomal abnormalities, gene upregulation or downregulation, somatic pathogenic variations, and hyper-methylation (Figure 1). Oesophageal cancer generally develops in response to a chronic inflammatory insult to the normal cells which results in somatic mutations, CNVs and chromosomal aberrations. These changes result in cancer formation and progression as normal epithelium undergoes basal cell hyperplasia and intraepithelial neoplasia (dysplasia) to give rise to invasive carcinoma [7,8,9].

Next-generation sequencing as a method to characterize genomic changes in cancer tissues offers the advantage of not having to have any prior knowledge of the DNA sequences being studied. NGS can be used to analyze the whole genome, whole exome or known cancer-causing genes using targeted cancer panels. These analyzes have detected widespread genomic alterations in OSCC [11]. The genomic characterization of tumors allows for specific molecular changes to be targeted for the development of unique therapies and interventions for each type of cancer, according to the underlying biological basis of each type of cancer. This allows scientists to use these genomic changes as biomarkers to monitor the drug response of the disease and identify the initiation and mechanisms underlying drug resistance. This information would allow for guided clinical decision making regarding the treatment of patients [5].

### 2.1. Gene Mutations

An initial large-scale whole-genome- and exome-sequencing analysis of OSCC performed in conjunction with comparative genomic hybridization identified somatic mutations in TP53. This was found to occur in 83% of OSCCs [12].

Compared to other solid tumors, OSCC has a high somatic mutation rate [13,14]. The most frequently mutated genes in OSCC include TP53, NOTCH1, PIK3CA, CDKN2A, CCND1, and FAT1 [15]. Other studies have also confirmed that the most frequently mutated cancer gene in OSCC was TP53. These mutations were the result of single-nucleotide variations and/or copy number losses, and this TP53 mutation is considered an early event in OSCC carcinogenesis [15,16]. Salem et al. [17] compared the molecular profiles of OSCC with oesophageal adenocarcinoma (OAC) and gastric adenocarcinoma (GAC) and found that OSCC showed lower mutational rates in ARID1A, KRAS, APC, PTEN, SMAD4, and CDH1. However, mutations were found to occur more frequently in KMT2D, SETD2, NOTCH1, RB1, CDKN2A, BAP1, FOXO3, and MSH6 compared with OAC and GAC. They also noted that OSCC exhibited higher expression of programmed death-ligand 1 (PD-L1) compared to OAC and GAC [17]. Moody et al. assessed the spectra of mutations that drive OSCC by identifying genes under positive selection based on the observed-to-expected ratios of nonsynonymous to synonymous mutations. They identified 38 genes, including known OSCC drivers such as TP53, CDKN2A, PIK3CA, NFE2L2 and NOTCH1 [16]. A summary of some of the published NGS studies in OSCC are summarized in Table 1.

Similar molecular epidemiology’s in OSCC exist across different population groups, with sub-Saharan African Asian (SSA)and North American OSCC patient cohorts showing similar genetic aberrations [2,16]. Despite the fact that OSCC is prevalent in the SSA region, there have been very few studies to characterize the genomic and transcriptomic landscape of the disease in this region. This is unfortunate, as such studies may help to reveal an underlying genetic cause for the high prevalence rates of the disease. In a study on Malawian patients, similar genes were found to be altered as those in other parts of the world. These altered genes include *TP53, CDKN2A, NFE2L2, CHEK2, NOTCH1, FAT1*, and *FBXW7* [2]. When a variety of high incidence areas were investigated, including SSA (Malawi), East Africa (Tanzania and Kenya) and Southeast Asia (China and Korea) and compared to a low-to-middle income, low-incidence country (Brazil) and high-income, low-prevalence countries (Japan and the UK), similar mutational profiles were found across all these countries. Genetic drivers that occurred at a higher rate in the African and Southeast Asian countries seem to be related to risk factors such as tobacco and alcohol. However, some were suspected of being germline variants [16]. Despite this similarity in the profiles of driver genes geographic regions and population groups, the SSA region is thought to have different or unique mutagenic mechanisms. There are thought to be three subtypes of OSCC in the SSA region that differ based on RNA and circulating nucleic acid sequencing [22]. Subgroups 1a and 1b had different levels of mutations in their CNAs, and had different frequencies of mutations in their *TP53*, and *TP63* genes, with group 1a showing higher levels of expression of genes involved in DNA replication, repair, and recombination. Subtype 2 was characterized by a higher expression of genes involved in neural differentiation [22]. 

The molecular profiles were divided into three classes by The Cancer Genome Atlas (TCGA) Research network. The TCGA network used a bioinformatic technique (iCluster) to group OSCC cases into 3 molecular subtypes. The NRF2 pathway is genetically altered in the first subtype, while mutations in the NOTCH1, ZNF750, KDM6A, KDM2D, PTEN and PIK3R1 genes characterize the second subtype. Finally, the Phosphoinositide 3-kinase (PI3K) pathway is disrupted in the third subtype [9]. Both the exposure to exogenous mutagens and endogenous processes have been found to give rise to distinct mutation patterns, known as mutational signatures [16]. Yang et al. [21] evaluated 24 surgically resected OSCC specimens by targeting deep sequencing examining single-nucleotide variations, indels, and copy number variations in 80 genes. A total of 115 genetic alterations were detected. The average number of genetic alterations was 4.9 per patient, as these changes were detected in 23/24 (95.8%) of patients. Any one or combinations of these genetic alterations could be targeted for the development of new therapies [21]. Table 2 illustrates the most frequently altered genes in oesophageal squamous cell carcinoma detected by next-generation sequencing.

### 2.2. Expression Changes

These signatures also differ when comparing human cancers and normal tissues [9]. An analysis of 20 tissue samples that were confirmed to be OSCC tissues and normal tissue from the same patient that was found adjacent to the cancer tissues and originating from India was performed using whole-genome oligonucleotide DNA microarrays. This analysis identified 881 upregulated genes and 1354 downregulated genes in OSCC. This study also validated three potential biomarkers OPN, ORAOV2 and FAP which were significantly overexpressed in OSCC. The study also identified several novel downregulated genes [24]. 

DNA methylation, histone modification, and loss of genome imprinting are all epigenetic changes that could lead to the development of OSCC [25,26]. Numerous genes have been shown to be hypermethylated in OSCC, including putative tumor suppressor genes like *CDKN2A/p16INK4a*. The hyper-methylation of these genes could serve as potential diagnostic or prognostic molecular markers for OSCC [3]. For example, CDKN2A is hypermethylated in 40–62% of OSCC cases. These cases are frequently at an advanced stage of disease and often lack CDKN2A expression [27]. Apolipoprotein B mRNA-editing enzyme catalytic polypeptide-like (APOBEC) is a DNA deaminase (cytidine deaminase)-inducing the conversion of cytosine to uracil, thereby regulating protein expression, innate immunity, and embryonic development. It is known to inhibit retrovirus replication and retrotransposon mobility. An analysis of 552 OSCC genomes from 8 different regions with different incidence rates found that, even though there was no association between the expression profile of APOBEC and OSCC incidence rates, they have often been associated with known OSCC risk factors. Specific APOBEC signatures are nearly always found in OSCC cases, suggesting that APOBEC-driven mutagenesis is an important and potentially absolute requirement for the development of most OSCC [16].

### 2.3. Genetic Drivers of OSCC

A better understanding of the genetic drivers of OSCC has been facilitated by the identification of mutational and expression profiles that are characteristic of OSCC or OSCC molecular subtypes. The molecules identified in these profiles has led to the identification of cellular pathways involved in OSCC signaling (Figure 1). Some of these pathways are commonly altered (upregulated) in many cancers, such as the WNT signaling pathway and signaling pathways initiated through the activation of various growth factor receptors (Figure 1) [19,28]. Alterations in these pathways all lead to altered phosphorylation of downstream molecule leading to changes in the activity of transcription factors and altered gene expression. These pathways depicted in Figure 1 may be upregulated due to other factors the leaf to the development of OSCC or may cause the disease themselves The FGF2-FGFR3 signaling axis promotes the progression of OSCC but is not thought to initiate the development of OSCC. This means that this pathway may be a good therapeutic target to controls disease progression and may be used as a prognostic marker for OSCC [29]. The expression of the neurofilament light chain (NEFL) protein is increased in OSCC, where it increases the expression of epidermal growth factor receptor (EGFR) promoting epidermal mesenchymal transition (EMT) and the progression of OSCC. e the EMT process of OSCC cells via the EGFR/AKT/S6 pathway, ultimately enhancing the invasion and migration of OSCC cells [30]. Tumor growth factor beta receptor III (TGFBR3) is frequently overexpressed in some tumors and is inked to the development of OSCC. High levels of TGFBR3 indicate a poor prognosis and may be a novel therapeutic target for OSCC treatment [31]. Constitutive activation of the Wnt occurs in OSCC [32] and the Wnt receptor, Frizzled (Fzd), has already been successfully targeted for therapeutic purposes [33]. The increased activation of the Wnt pathway results from mutations in APC and Axins [32]. These molecules could, therefore, be suitable for targeted treatments. 

**Figure 2:**
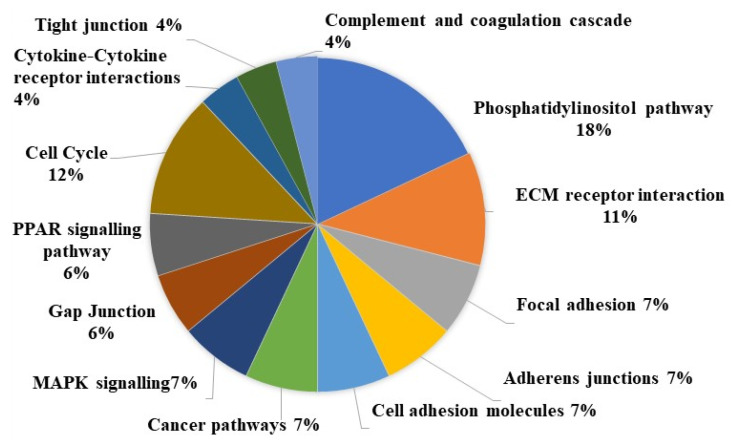
The most prevalent pathways affected in OSCC. This figure depicts the most prevalent pathways affected by molecular/genomic changes in OSCC. This is assessed by the number of genes in each pathway whose expression is altered. This alteration may be up- or downregulation. By grouping the affected genes by the pathways, they are involved in the most common pathways affected are the PI3K/AKT/mTOR pathway (*PIK3CA*, *PTEN*), the cell cycle regulation pathways (*TP53*, *CDKN2A*, *RB1*, *CREBBP*), ECM receptor interactions [36].

The most commonly affected pathways involved in OSCC signaling are shown in Figure 2. Cell cycle pathways are commonly affected, where cell cycle suppressor genes are often deleted, leading to cell proliferation. These include genes such as CDKN2A and RB1 (Retinoblastoma protein). At the same time, cyclins like CCNE1, CCND1, CCND2, CDK4 and CDK6 are commonly overexpressed (Reviewed in [34]). This explains why 12% of all genes whose expression is altered are found in cell cycle associated pathways (Figure 2). The PI3K pathway is altered in in 59% of OSCC samples in the TCGA. Some of the proteins that are involved in activating the PI3K pathway include EGFR, FGFR1 and PIK3CA, while proteins that can suppress the PI3K pathway include PTEN and PIK3R1 [9]. The course and clinical outcomes of OSCC are influenced by these driver mutations, as they can decide the aggressiveness of the tumor, how the tumor responds to treatment and drug resistance. These driver mutations may also serve as targets for therapy [9]. This explains why 18% of all genes whose expression is altered in OSCC play a role in this pathway, and it is the most affected pathway (Figure 2). The other pathways that are affected in OSCC relate to the common hallmarks of cancer and processes such as metastasis, ECM receptors, gap junctions and adhesions, and general cancer related pathways such as resistance to apoptosis signals (Figure 2).

Those genes that therefore can be considered as drivers of OSCC, by mutation or expression change, include TP53, CDKN2A, PIK3CA, NFE2L2, NOTCH1 and APOBEC amongst others. However, attempts to target these genes for theopoetic purposes has not always been successful [35].

## 3. Tumor Mutational Burden

Cancer is the phenotypic endpoint of accumulated genetic and epigenomic alterations. A metric was created to quantify the number of mutations present in a tumor. Metric is known as the tumor mutation burden (TMB) [37] and is calculated by dividing the number of missense mutations in the tumor genome by the size of the of the DNA being measured, in megabases. Normally this is by either the size of the protein coding genes which is 35–45 Mb in humans [38], or by the entire genome, 3.3 Gb in humans. The former is used for whole exome sequencing (WES) and the latter for whole-genome sequencing (WGS) [39].

The TMB is also expressed as the mutations per million bases of the tumor, genome examined [16,40]. TMB is a continuous variable, ranging from 0.001 mut/Mb to more than 1000 mut/Mb as has been observed across and within cancer types [38]. It is speculated that a tumor bearing a higher TMB level may be highly immunogenic as it is likely to harbor more neoantigens. These neoantigens can then be targeted to a higher degree by activated immune cells capable of inducing an anti-tumor immune response, which would result in a good clinical prognosis [37,41]. This increase in neoantigens occurs because TMB is believed to be a good indicator of the number of immunogenic neopeptides displayed on the surface of tumor cells, which influences patient response to ICIs [38].

The Checkmate trials are a clinical trial of the adjuvant checkpoint inhibitor Nivolumab in high-risk bladder cancer that has invaded muscle-tissue [42]. According to the Checkmate trials, allowed the researchers to establish a TMB threshold for a high TMB is ≥10 mutations per megabase (10p6) (mut/Mb) and was demonstrated as a robust, independent biomarker of response based on the objective response rates of the tumors in those studies not improving much beyond this threshold [37,39]. Although TMB—H prevalence varies widely between different types of tumors, it has emerged as a potential biomarker for tumors that are likely to respond to immune checkpoint inhibitor therapy [43].

In oesophageal cancer, it was found that clinical factors, such as age, gender, tumor grade, tumor stage, race, and prior radiation treatment, were not associated with TMB levels and there was no significant correlation between TMB and TNM stages. A multivariate regression analysis of TMB levels and OSCC associated risk factors, indicated that TMB is a risk-independent prognostic factor, with a lower TMB being associated with a better prognosis [37]. Oesophageal adenocarcinoma (OAC) has a higher TMB than OSCC. A study comparing the two have found a mean TMB-High >17 mut/Mb in 3% of OSCC were compared to 8% for OAC [7,38].

## 4. MicroRNAs

MicroRNAs (miRNAs) are 20–24 nucleotides long and are therefore considered to be small non-coding RNAs. They are also well-conserved, and function to regulate the translation of mRNAs. As such they are post-transcription gene regulators involved in cell death, proliferation, and differentiation [44]. Hundreds of genes are regulated by miRNAs. Gene regulation occurs through the miRNA binding to the 3’-untranslated region (3′-UTR) of the target mRNA. This can lead to decreased gene expression through the degradation of the mRNA or by inhibiting translation. More than half of all miRNA coding regions are located in fragile sites and regions of the genome that are associated with cancer development in the human genome [45]. Additionally, miRNAs can act as oncogenes to promote the development and progression of cancer, or they can act as a tumor suppressor that impedes the development of tumors [46]. They have emerged as potentially significant diagnostic and prognostic biomarkers of oesophageal cancer. Potentially miRNAs can play a causal role on cancer development since they can function as oncogenes or tumor suppressors [44]. Abnormal expression levels of different miRNAs can be detected from tumor tissue and compared to the expression levels of adjacent normal tissue. Aberrant miRNA expression is commonly found in many human disorders including cancer. This method of transcriptional regulation is of interest in cancer research as it plays a role in cancer progression. 

The current advances in elucidating the role played in OSCC by miRNAs are due to new technologies that facilitate miRNAs profiling. These technologies have led researchers to explore the great potential miRNA profiles have as diagnostic and prognostic biomarkers for OSCC. These miRNAs profiles may also serve as lead targets for new treatments [20]. The miRNA expression profiling of OSCC is distinct from that of oesophageal adenocarcinoma [41]. Some studies analysing the expression profiles of miRNA using unsupervised hierarchical clustering demonstrated that these profiles can be used to classify different types of oesophageal cancer due to the significant differences in the miRNA profiles between these oesophageal disease groups [44]. A polymorphism in miRNA 3184 has been detected in black South Africans. This polymorphism, named rs6505162 and the rs6505162 SNP, is located in the overlap of two oppositely orientated miRNAs, miR314 and miR413. This allows it to affect both these miRNAs as well as the gene coding for NSRP1. The presence of this polymorphism resulted in increased associated risks for developing OSCC [24].

In a study of Chinese men with OSCC, Bai et al. took samples from the tumor and from adjacent normal tissues. They found that miR-1269 had an altered expression pattern unique to cancer tissues. The transcript levels of miR-1269 was higher in tumor samples (*p* < 0.001,). This was confirmed using OSCCS cell lines; Eca-109, KYSE-150, TE-10 and TE-1; where transcription levels of miR-1269 were also found to be higher than the levels in the normal oesophageal endothelial cell line, Het-1A (*p* < 0.001) [46].

**Figure 3:**
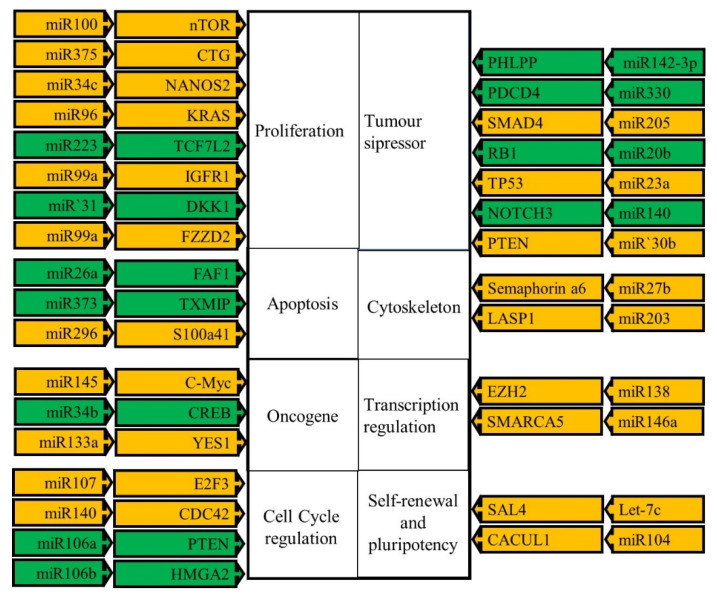
The targets of miRNAs with altered transcript levels in OSCC. The miRNAs are in the outer block arrow pointing towards the gene they target for translational regulation. The hallmark of cancer these genes are involved in is represented by the large blocks in the center of the figure. The miRNAs either affect the expression of this gene positively or negatively thereby contributing to OSCC development and progression. The miRNA gene blocks in orange signify that the miRNA acts as a tumor suppressor inhibiting the cancer process in the indicated block, while those marked in green act as oncogenes, enhancing the cancer activity indicated in the block [21,44,45,48,60,61,63,76,83,86].

The importance of miR-1269 expression in OSCC was also demonstrated. Overexpression of this miRNA was associated with lymph node metastasis, and advanced TNM stage (*p* = 0.008). It is likely that this miRNA, therefore, plays an oncogenic role in the development and progression of OSCC. The expression profile of miR-1269 may therefore be used as an independent prognostic biomarker for OSCC [46]. Tumor-derived miRNAs are not degraded by endogenous ribonucleases, and are therefore present in circulating human blood (serum and plasma) in a remarkably stable form; their expression levels are consistently altered among different individuals, and are present at levels that can be detected and measured and can therefore be used as biomarkers for the detection of tumors [47]. Mitchel et al. in their study of miRNA in prostate cancer patients found that serum levels of miR-141 in prostate cancer patients was elevated, demonstrating that a tumor-expressed miRNA can be used to diagnose cancer with a high degree of both sensitivity and specificity [48]. Table 3 show some examples of miRNAs that can be targeted as biomarkers in oesophageal cancer [44]. These include diagnostic biomarkers, prognostic biomarkers and finally biomarkers that can be used as predictors for the response to treatment. A list of these miRNAs is given in Table 3. The targets and pathways these miRNAs target, are depicted in Figure 3.

## 5. Alternative Splicing

Alternative splicing is a tightly regulated process that involves the production of a variety of alternatively spliced transcripts that allows for the expression of multiple protein isoforms from a single gene. Therefore, alternative splicing increases the protein diversity and increases the catalog of proteins encoded for by the same number of genes [87]. Approximately 94% of all human genes contain introns that are alternately spliced. Alternative splicing and its dysregulation have been reported to play a role in the development and progression of OSCC [88]. This is due to the numerous pathologies being caused by dysregulated splicing. The ability of alternate splicing to contribute to development and progression of cancer is due to splicing promoting the loss of function of tumor suppressor genes or causing increased oncogene activity and the activation of pro-oncogenic pathways [89]. Splicing switches are also activated in tumors. This involves the splicing pathways favoring the splicing of mRNAs to form a pro-oncogenic isoform. These switches may favor isoforms that can promote the development of cancer by increasing growth and proliferation, invasion and metastasis and immune evasion. They can also contribute to the resistance of the cancer to drugs being used to treat it [89,90,91,92].

**Figure 4:**
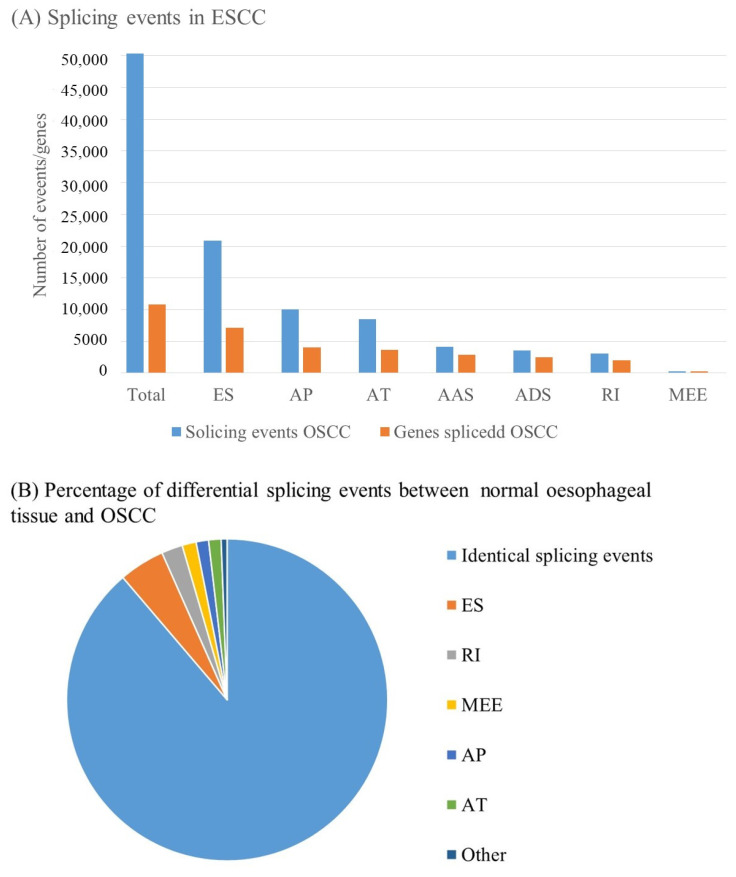
Frequency of different splicing events. (**A**) This is a representation off the frequency of the seven types of AS involved in OSSC as detected by Sun et al. [75]. Exon skipping (ES) is shown to be the most prevalent, affecting the highest number of genes. This is followed in decreasing order of frequency by alternat promoter (AP) events, alternate terminator (AT) events, alternate acceptor sites (AAS), alternate donor sites (ADS). Intron retention (RI) events and finally events involving mutually exclusive exons (MEE). (**B**) Percentages of differentially spliced AS events between OSCC clinical samples and matched normal samples with fraction of each type of splicing event [93].

Sun et al. performed a systemic analysis of integrated AS events in 185 OSCC patients from the TCGA. They used the online tool SpliceSeq to identify a total of 50342 AS events in 10766 genes, which included 20843 exon skips in 7174 genes, 10033 alternate promoters in 4046 genes, 8448 alternate terminators in 3690 genes, 4145 alternate acceptor sites in 2871 genes, 3590 alternate donor sites in 2463 genes, 3038 retained Introns in 2001 genes, and 245 mutually exclusive exons in 237 genes; these results demonstrating that, among the seven types of AS events, exon skipping was the main splicing pattern while mutually exclusive exon splicing was the least frequent event in OSCC patients (Figure 4) [89].

**Figure 5:**
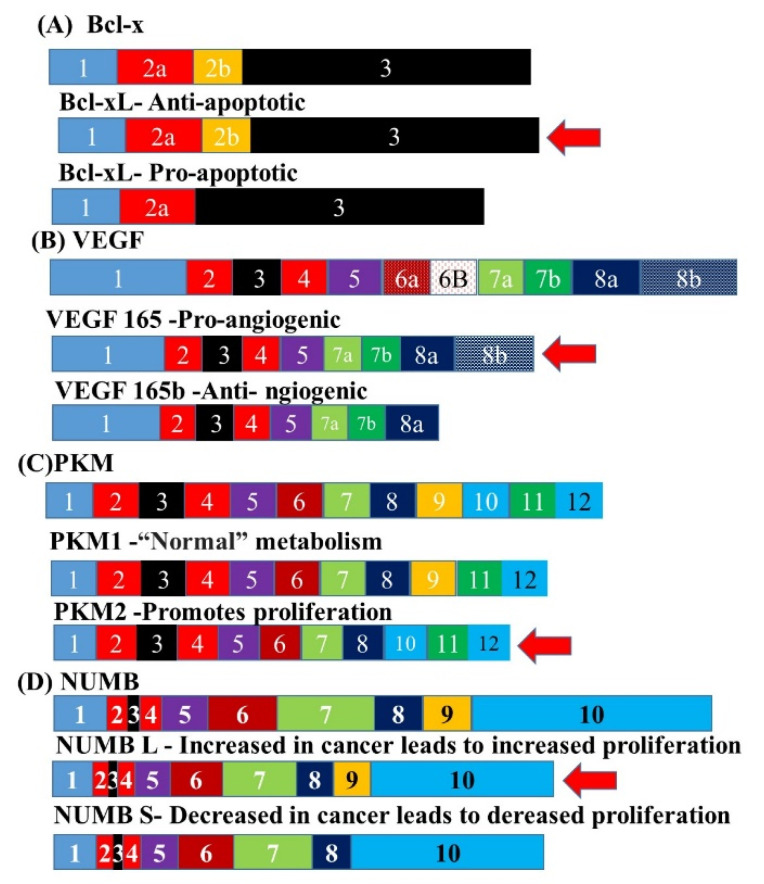
The role of alternative splicing of specific genes in tumorigenesis. The diagram illustrates the roles of different isoforms of Bcl [96] (**A**), VEGF [97] (**B**), PKM [96] (**C**) and NUMB [98] (**D**) in specific hallmarks of cancer such as resistance to apoptosis (Bcl), uncontrolled proliferation (NUMB), a metabolic shift to glycolysis (PKM) and increased angiogenesis (VEGF). The red arrows indicate the isoforms that are found to be expressed at higher levels in OSCC. The same exons in the same genes are shown by identical colors.

Xie et al. analyzed survival-associated alternative splicing events using the records of 165 OSCC patients consisting of 83 OAC and 82 OSCC patients from the TCGA database and 2199 events in OSCC were found to be significantly associated with survival. Prognostic models were built for each AS event and combined AS events in OSCC and its histological subtypes. Most of the models showed satisfactory predictive efficacy for the survival of patients [94]. Splicing events related to the hallmarks of cancer account for most of the oncogenic splicing events in OSCC. These include the increased expression of isoforms involved in increased proliferation, altered cell junction, and increased cell migration. Figure 5 illustrates how alternately spliced isoforms could contribute to both cancer development and progression. Some of these different isoforms can be targeted as potential biomarkers for early cancer detection and diagnosis and as targets for novel therapeutics [95]. Examples of the targetable splice variants are listed in Table 4.

By comparing the alternative splicing between healthy oesophageal tissue and oesophageal cancer tissue, a study was able to quantify changes in the number of genes affected by splicing events and the frequency of the types of splicing events. This study made use of the mixture of isoforms (MISO) probability framework to identify splicing changes that occurred [94]. This was confirmed by comparing splicing events between OSCC cell lines and normal oesophageal cell lines. Approximately 32,891 splicing events were identified in normal cell lines and 2.8% of these splicing events were altered in the OSCC cell lines [94]. Another study analyzed the splicing events that are characteristic of OSCC cancer patients by examining the difference in splicing between cancer and healthy oesophageal tissue from 79 OSCC patients. This study identified 2326 AS events in 1738 genes that were differentially spliced in OSCC tissue compared to healthy oesophageal tissue. Of these alternately spliced genes, 1360 genes were predicted to be significantly associated with overall oesophageal cancer patient survival [95].

Oncoviruses can contribute to tumorigenesis by changing the regulation of splicing. These oncogenic viral transcripts are spliced to give rise to protein isoforms that promote cancer development and progression. Oncoviruses also promote the growth of cells by altering cellular pathways that also promote the altered expression of splicing factors.

## 6. Viral Oncogenesis

All cancers arise because of somatically acquired changes in the DNA of cells that lead to them to become cancer cells. The various changes have become known as the hallmarks of cancer [107]. These changes to the DNA may be the result of the acquisition of completely new DNA sequences from exogenous sources. Commonly the sources of these new sequences of DNA are the result of viral infection. Most notably viruses such as human papillomavirus (HPV), Epstein Barr virus (EBV), hepatitis B virus (HBV), human T lymphotropic virus 1(HTLV-1) and human herpesvirus 8 (HHV-8), each of which is known to contribute to the development and progression of various cancers [108]. It is estimated that up to 10% of all human cancers are caused by infection with oncogenic viruses and the number of cancer cases that are attributable to viral infections is much higher in developing nations where 22.9% of all cancers are the result of infection with oncogenic viruses, compared to 7.2% in the developed world [109].

In South Africa it is estimated that 13.5% of the population are infected with human immunodeficiency virus (HIV), meaning that approximately 7.97 million in 2019 are classified as people living with HIV (PLWHIV) [110]. According to UNAIDS, in 2018 about 62% of people living with HIV in South Africa were on treatment and 54% were virally suppressed. This translates into increased life expectancy for a significant number of HIV-positive persons. This has also led to an ageing HIV-positive population. There is a concern that the incidence of non-AIDS-defining cancers (NADCs) and resulting mortality may increase in this group, as cancer is a disease that is associated with age and these patents will be immunocompromised [111]. As such, PLWHIV have a higher risk of developing certain NADCs compared with the general population. The effect of HIV on the immune system may lead to a decrease in the ability of the immune system to control oncogenic infections and an increase in co-infections with hepatitis and human papillomavirus [9]. These increases in co-infections occur regardless of treatment with anti-retroviral therapy (ART). Mechanistically, HIV infection increases the risk of developing cancer and contributes to disease progression that is associated’ with B-cell, T-cell, and NK-cell dysfunction, persistent inflammation, and mucosal epithelial abnormalities. These include multiple types of cancer. HPV-associated malignancies progress rapidly. These two viruses, the human papillomavirus (HPV) and human immunodeficiency virus (HIV), although substantially virologically different, complement and foster each other in many aspects [112].

Several studies looking at HPV prevalence and its association with squamous cell cancers from different anatomical regions have been conducted using various methods of tissue analysis. These included studies of the prevalence of HPV in oesophageal squamous cell carcinoma (OSCC) and studies examining any possible causative effect HPV infection may have on the development and progression of OSCC. These studies that failed to detect HPV in OSCC patients, were performed in populations from countries with a lower HPV incidence such as the United States and European nations. These studies performed in countries with a higher incidence of HPV are more likely to find a significantly higher percentage of HPV in oesophageal cancer [113]. Apart from differences in the populations examined, these differences may be due to different methodologies used. However, Wang et al. in their study of 435 patients from multiple regions in China and the USA, found that HPV DNA is commonly found in oesophageal cancer and is independent of geographic region and the ethnicity of tested subjects [113].

Serologic analyzes of HPV in oesophageal cancer patients are inconclusive, most likely due to these studies not distinguishing between co-incidental HPV infection at different body sites as the presence of HPV may be different in other body sites compared to HPV infection in the oesophageal squamous epithelium [12]. Consequently, the more reliable studies have been those involving tumor tissue biopsy analysis with DNA extraction. Whilst several studies have looked at the role or influence of HPV in OSSC, there is a paucity of data when it comes to the possible compounding effect of HIV and HPV in OSCC. The molecular hallmarks associated with latency and persistence of HIV and HPV infection in oesophageal cancer are not yet understood.

Despite the important contribution HPV infection has on the development of cancer in various tissues, HPV oncoproteins may influence different molecular pathways depending on the tissue they are expressed in, due to tissue-specific factors [8]. However, regardless of the cancer type, the HPV oncoproteins E6 and E7 are the primary HPV proteins that mediate cellular transformation [114]. These proteins enhance cell proliferation and promote the development of new oncogenic mutations due to the associated loss of genomic stability. The E6 protein can mediate the degradation of p53 and its ability to induce telomerase expression. The E7 HPV protein contributes to transformation by promoting the degradation of the retinoblastoma (Rb) family of proteins [114]. The oncogenic activities of the HPV18 E6 and E7 in oesophageal (EC109 and EC9706) and tongue (Tca83) cancers was studied using based on cell lines. The study demonstrated that E6 downregulated p53 and its downstream target p21 in a similar manner, whilst p130 was preferentially targeted by E7 in oesophageal cell lines [8].

Cao et al. tested 105 OSCC specimens with in situ hybridization and confirmed 29 HPV positive cases, with all cases being HPV-16 positive. They also found that the HPV positive patients had statistically better overall survival rates with a 63% reduction in risk of death. This resulted in the use of HPV status as an independent prognostic marker for patient survival [115].

HIV and HPV infections were found to improve the overall survivability of head and neck squamous cell carcinoma patients. A clue as to the underlying cause of this increased survivability was that patients with high expression levels of TP53 tended to have lower survival rates (P 1⁄4 0.20). An analysis of TP53 expression in HIV and HPV patients showed that high TP53 expression in HIV-positive patients was significantly associated with decreased survival (P 1⁄4 0.04). This was not observed in the HIV negative patients [8].

Features of tumors that can be detected and differentiated through histological techniques to diagnose cancers, have been found to not be able to distinguish tumors of the upper gastrointestinal tract [17]. For instance, in terms of their underlying molecular causes and profiles, there is a large degree of similarities between OSCC and head and neck squamous cell cancer (HNSCC) which are very distinct from oesophageal adenocarcinoma [17]. Tumors that are the result of viral infection, such as Epstein Barr Virus (EBV), or Human Papilloma Virus (HPV) express neoantigens that are the products of the viral open reading frames [38].

## 7. Treatment of OSCC and the Clinical Application of Molecular Profile Data

The clinical applications of the data gathered through the identification of the genomic, transcriptomic and proteomic changes that accompany the development and progression of OSCC are varied. Perhaps the most obvious is the identification and adoption of diagnostic and prognostic biomarkers [77]. Prognostic biomarkers that have been identified to be useful in OSCC include the *MIB-1* gene which is an indicator of cell proliferation. Increased expression of this gene is generally an indicator of poor prognosis [116]. Proteins in the p53 G1-S transition signaling pathway, including P53, P21 and CIP1 can all be used as prognostic markers in OSCC, with increased expression of these proteins indicating a good prognosis and 5-year survival chances [117]. However, the increased expression of genes associated with chemoresistance is an indicator of poor prognosis these include *ts1, ercc1* and *gstp1* [118].

These biomarkers can also be used to guide treatment choice and monitor treatment response. For instance, the expression levels of *p53* and *14-3-3 sigma* can give an indication of the response of a tumor to chemoradiation therapy, (CRT) with the therapy being less effective in OSCC patients that are p53 negative an 14-3-3 sigma positive [119]. At the same time OSCC patients that were p53-positive and MT-positive respond poorly to CRT. Increased expression of the cell cycle marker CDC25B is associated with a positive outcome following CRT treatment [120]. The response to CRT can also be predicted through establishing the expression level of the proliferation marker KI-67 [121] and by increased activity of the Hedgehog signaling pathway which indicates a good response to CRT and an increased 5 year survival [122]. Due to the higher somatic mutation rate observed in OSCC [14], the tumor mutation burden (TMB) is a risk- independent prognostic factor in oesophageal cancer [16,40]. A higher TMB level occurs in tumors, resulting in a better anti-tumor immune response and a better prognosis [23,37]. Although the prevalence of TMB- High varies widely between different types of tumors, it is a reliable indicator of treatment response [37,39,43].

In addition to these roles another clinical application of the molecular landscape of OSCC is the use of this know; edge to develop targeted therapies [77]. These would be based on t targeting cellular pathways that play a role in OSCC and therefore, suitable targets for new therapies. Surgery, which is currently the mainstay of treatment for oesophageal cancer, is associated with high morbidity and even successful esophagectomies can result in decreased health-related quality of life, eating difficulties and malnutrition [123]. A better understanding of the genetic drivers of OSCC as well as the identification of mutational signatures, molecular subtypes, and expression profiles of OSCC has resulted in the identification of cellular pathways and specific genes suitable for targeted treatments. Some of these pathways that could be targeted include the Cell Cycle pathway by targeting genes such as CDKN2A, RB1, CCNE1, CCND1, CCND2, CDK4 and CDK6 [88,89].

Since over 50% of all miRNA genes are found in genomic regions associated with cancer or in fragile sites in the human genome [45], altered, pathogenic miRNA expression is commonly found in many human disorders including cancer. Abnormal expression levels of different microRNAs can be detected in tumor tissue [20]. Their diagnostic potential is increased by the fact that tumor derived miRNAs are resistant to endogenous ribonuclease activity so they can be present in circulating human blood [20,44,47]. Some studies analyzing the expression profiles of miRNA using unsupervised hierarchical clustering showed significant alterations in miRNA expression profiles in multiple types of oesophageal diseases. These changes in expression can then be used to classify these diseases [44]. Increasingly miRNAs are being examined for their use as therapeutic targets. Different strategies are being used to do this. One involves the use of synthetic antagonists or inhibitors to neutralize oncogenic miRNAs. This is known as mRNA suppression or replacement therapy. This can also be accomplished using oligonucleotides to mimic, miRNAs (agomirs) [124,125]. Additionally, the injection of miRNAs directly into a tumor has been shown to have therapeutic effects. Subcutaneous injection of miR-375 an miR-27a suppressed OSCC growth [86,126].

In cancer, aberrant alternative splicing can promote splicing switches between pro- and anti-oncogenic variants. These switches can lead to the promotion of cell growth, drug resistance, changes in cell adhesion and migration, metastasis, and immune escape [89,90,91]. Splicing can also lead to the production of inactive versions of tumor suppressor proteins or even the activation of oncogenes and cancer pathways [45]. Some of these different isoforms can be targeted as potential biomarkers for early cancer detection and diagnosis and as targets for novel therapeutics [95]. The changes that can occur in alternative splicing that contribute to the development and progression of OSCC can also be targeted. Currently there are no therapies for OSCC that target alternative splicing, but this process can easily be targeted in several ways. These include using small molecular regulators of splicing factors or splicing regulatory proteins [127]. Finally, the therapeutic regulation of alternate splicing is through the use of oligonucleotide-based therapies that target oligonucleotides that can also be used to target splicing. These are small nucleotides that are designed to have complementary sequences to mRNAs, resulting in them hybridizing to the target mRNA and alter splicing [128].

## 8. Conclusions

**Figure 6:**
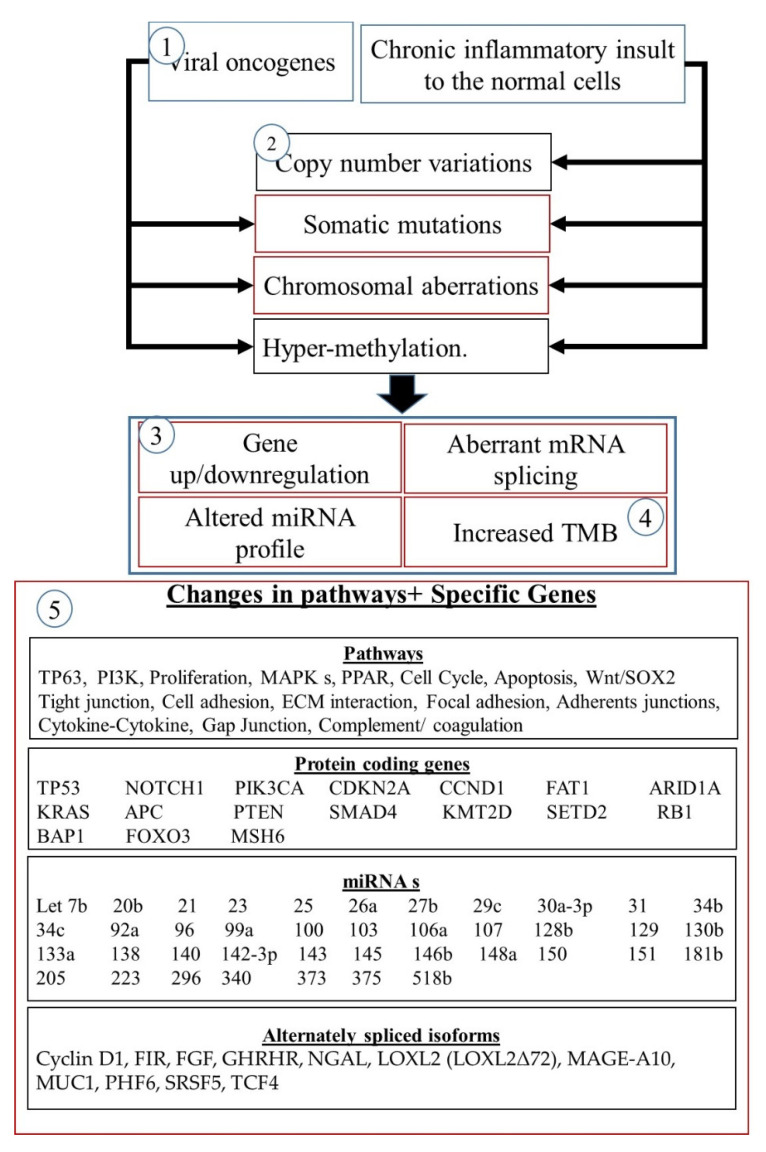
The genomic and molecular changes associated with OSCC. Following genetic damage from viral infection or in the case of OSCC damage to the tissue lining the oesophagus changes can occur at the genetic level relating to somatic mutations, copy number variation, chromosomal aberrations, or epigenetic changes such as hyper-methylation. This can result in changes in the expression patterns of genes or miRNAs as well as changes in the patterns of mRNA splicing. It can also lead to an increased mutational burden. These changes can be used as biomarkers in multiple ways. (1) Infection with oncoviruses serves as indicator for further screening. (2) The presence of patterns of genetic abnormalities can be used as biomarkers. (3) Profiles of alternate transcripts, gene expression, miRNAs used as biomarkers. (4) Tumor mutational burden can be used as a prognostic tool. (5) The presence of specific genes or increased expression of genes involved in certain pathways can be used as a biomarker.

The knowledge gained through the deciphering of the biological basis of OSCC through genomic characterization can be used to identify targets for the development of new drug therapies, as well as new biomarkers for various roles in the management of OSCC. These biomarkers can serve as diagnostic or prognostic biomarkers, or they can be used to monitor the response of the cancer to drugs or other treatments. This data can be used to assist the oncologist in selecting the correct treatment for a specific patient ^5^. Identification of diagnostic biomarkers that can detect cancer early is essential. This is demonstrated by the difference in the 5-year survival rate between oesophageal cancer at the early and late stages following treatment, with 90% of patients treated at the early stage surviving past 5 years compared to only 6% ~ 15%. for those patients diagnosed at the middle or late stages. The fact that most oesophageal squamous cell carcinoma cases are normally only detected at the late stages of the disease, especially in the developing world, means that these diagnostic biomarkers that could lead to the early detection of OSCC could make a huge difference in the management of this disease. New therapeutic targets can lead to the development of new more effective therapies which promises to improve the long-term survival and quality of life for OSCC patients. For instance, some known genetic drivers of OSCC include TP53, CDKN2A, PIK3CA, NFE2L2 and NOTCH1 [12,15,16] can serve as lead targets for the development of new treatments (Figure 6, Table 5).

Figure 6 demonstrates the use of various target profiles, pathways, genes and genetic aberrations as biomarkers. The advent of innovation and improvements in, the field of genomics heralds a new era in the management of cancer by allowing us to identifying potential biomarkers for early detection and better application of precision oncology, which should surely result in improvements in mortality and morbidity associated with esophageal squamous cell cancer. Table 5 lists the molecules that can be used in the clinical setting to manage OSCC.

In summary studies into the molecular landscape of OSCC can assist in the management of the disease by providing clinicians with the tools for more effective screening, diagnosis and prognosis and can allow for the development of new therapies as well as for the more effective use and targeting of existing therapies. However, it is important that these molecules and the pathways they act in are well characterized as they may not function in these roles to the same extent in every population group or even individual. This is also why it is important to develop and use multiple biomarkers. Variations amongst individuals is also why any diagnosis, prognosis or treatment decision made using these molecules must rely on information from more than one source. In other words, these tests must be made up of arrays that check the expression or mutation status of multiple biomarkers. This is also why signaling pathways are very promising, as here the levels or status of multiple components in the pathway can be assayed to come to a conclusion.

## Figures and Tables

**Table 1:** NGS studies of oesophageal squamous cell carcinoma [8].

Method	Sample Number	Number of Non-Silent MUTATIONS/ Tumor	Ref.
Whole exome sequencing	12	83	[18]
Whole exome and transcriptome sequencing	20 exomes and 119 transcriptomes	59	[19]
			
Whole exome sequencing	113 exomes	82	[20]
Whole genome and exome sequencing	14 genomes, 90 exomes	104	[21]

**Table 2:** The mutational profiles of important genes in OSCC as established in various studies.

Gene Symbol	Chromosomal Location	Nonsynonymous Mutation	Copy Number Loss	Copy Number Gain	Ref.
*TP53*	17p13.1	92	0	0	[18]
		83	0	1	[20]
		60	0	0	[19]
		93	0	0	[20]
		88	0	0	[15]
*NOTCH1*	9q34.3	33	0	0	[18]
		9	0	0	[20]
		8	0	0	[19]
		14	0	0	[20]
		19	0	0	[15]
*PIK3CA*	3q26.3	0	0	0	[18]
		5	4	0	[20]
		7	10	0	[19]
		9	2	0	[20]
		17	0	0	[15]
*CDKN2A*	9p21.3	8	0	0	[18]
		5	0	44	[20]
		3	0	33	[19]
		8	0	12	[20]
		8	0	64	[15]
*CCND1*	11q13	0	0	0	[18]
		0	46	0	[23]
		0	46	0	[19]
		0	33	0	[20]
		0	64	0	[15]
*FAT1*	4q35.2	8	0	0	[18]
		5	0	0	[23]
		12	0	0	[19]
		11	0	0	[20]
		15	0	0	[15]

**Table 3:** miRNAs that can be used as biomarkers in oesophageal cancers.

miRNA	Type of Marker	Host Gene or Chromosome Location	Description	Ref
	Upregulated			
	**Changes are a consequence of OSCC**			
142-3p	Prognostic	GRCh37	Indicates poor prognosis.	[49]
181b	Prognostic	SALL4	Indicates poor prognosis.	[47]
223	Prognostic	Xq12	Indicates poor prognosis.	[50]
146b	Prognostic	10.	Indicates poor prognosis.	[51]
	**Changes contribute to OSCC development ad progression**			
20b	Diagnostic	-	Targets RB1 and TP53.	[52]
21	Diagnostic: and prognostic	TMEM49	Found in serum in serum and plasma indicates poor prognosis. Target for inhibition.	[53]
23	Prognostic	19.	Indicates poor prognosis.	[54]
25	Diagnostic	MCM7	Plays a role in metastasis. Target for inhibition.	[55]
34b	Diagnostic	11q23.1	Oncogenic role in OSCC. Target for inhibition.	[56]
96	Prognostic	MIRN183-MIRN96-MIRN182 cluster 7q32.2	Indicates poor prognosis. Target for inhibition.	[57]
128b	Prognostic	3p22	Indicates poor prognosis. Target for inhibition.	[58]
129	Diagnostic and prognostic	11p11.2	Indicates poor prognosis.	[59]
130b	Diagnostic	2q11.21-q11.22.	Promotes angiogenesis. Target for inhibition.	[60]
138	Diagnostic			[61,62]
151	Diagnostic	FAK	Oncogenic.	[45]
330	Diagnostic	724063	Oncogene. Target for inhibition.	[63]
373	Diagnostic	Linked to MIRN371 and MIRN372	Promotes migration and invasion. Target for inhibition.	[64]
	**Down-regulated**			
	**Changes are a consequence of OSCC**			
27b	Prognostic	9q22,	Indicates poor prognosis.	[54]
103	Prognostic		Indicates good prognosis.	[65]
34c	Diagnostic	11q23.1	Tumor suppressor.	[59]
140	Diagnostic	16q22.	Downregulated.	[66]
143	Predictive and prognostic	5q33	Indicates a poor prognosis and predicts nonresponse to treatment.	[67]
145	Diagnostic and predictive	5q33.1	Predicts poor response to treatment.	[67]
205	Diagnostic and Predictive	1q32.2	Downregulation serves as diagnostic marker. Upregulation predicts poor response to treatment.	[65]
518b	Prognostic	19q13.42	Indicates poor prognosis.	[68]
	**Changes contribute to OSCC development ad progression**			
26a	Prognostic	CTDSPL	Indicates poor prognosis. Possible therapy through mimics.	[69]
29c	Diagnostic	1q32.2	Leads to increased proliferation. Possible therapy through mimics.	[70]
30a-3p	Diagnostic	6q13	Downregulation leads to increased proliferation.	[71]
31	Prognostic and Diagnostic	9p21.3	Found in serum indicates poor prognosis.	[72]
92a	Prognostic and predictive	C13ORF25	Indicates good prognosis and predicts nonresponse to treatment.	[73]
99a	Diagnostic	MIR99AHG	Tumor suppressor. Possible therapy through mimics.	[74]
100	Diagnostic	MIR100HG	Downregulated. Possible therapy through mimics.	[75]
106a	Prognostic and predictive	Xq26.2	Indicates poor prognosis and predicts nonresponse to treatment.	[76]
107	Prognostic	10	Indicates poor prognosis. Possible therapy through mimics.	[77]
133a	Diagnostic	MIB1	Tumor suppressor.	[78]
133b	Diagnostic	LINCMD1	Tumor suppressor.	[78]
148a	Prognostic and predictive	7p15.2	Downregulation is an indicator of poor prognosis and predicts lack of response to treatment.	[79]
150	Prognostic	19q13.33	Downregulation is associated with poor prognosis.	[80]
203	Diagnostic	14q32.33	Tumor suppressor. Possible therapy through mimics.	[81]
296	Prognostic	MIRN296	Downregulation indicts poor prognosis. Possible therapy through mimics.	[82]
340	Diagnostic	16q11	Acts as a tumor suppressor. Possible therapy through mimics.	[83]
375	Plasma Diagnostic, prognostic	DLK1 and DIO3	Decreased level in plasma indicates poor prognosis. Possible therapy through mimics.	[84]
Let-7d	Diagnostic	387247	Blocks EMT transition low levels indicates poor prognosis.	[85]

**Table 4:** Splice variants involved in OSCC.

Gene	Function	Splicing	Ref.
Cyclin D1	Proliferation	Cyclin D1b levels increased.	[99]
FIR	Splicing, apoptosis, and transcription	Increased expression of isoforms lacking exon 2.	[100]
FGF	inhibits proliferation	Splice variants of FGF-2 and variant b increased in cancer.	[101]
GHRHR	Growth hormone receptor	Splice variant 1 levels increase.	[102]
LCN2, NGAL	Inhibits proteolysis	Expression of NGAL-2 and NGAL-3 increased.	[103]
LOXL2	ECM remodeling	LOXL2Δ72, which lacks 72 promotes greater cell migration.	[104]
MAGE-A10	Development	Additional exons 3A and 3B.	[105]
MUC1	Cell adhesion	MUC1/C, D, and Z are expressed at higher levels.	[106]
PHF6	Transcriptional regulation	Splice variants retaining introns overexpressed.	[93]
SRSF5	Splicing factor	Different splice variants have different splicing regulatory functions.	[93]
TCF4	WNT signaling	Unique isoforms isolated from various cancers.	[93]

**Table 5:** Viable genes, pathways, isoforms and miRNAs that can be targeted for the development of clinically useful biomarkers, or could be used as lead targets for new therapies.

Gene Mutations/Expression	
Methylation status	*CDKN2A/p16INK4a*
Somatic mutations present in OAC, gastric cancer and OSCC	TP53, *PIK3CA, CDKN2A, CCND1, ARID1A, KRAS, APC, PTEN, SMAD4, NFE2L2, CDH1* and *FAT1*
Mutations unique or more common in OSCC	*KMT2D, SETD2,CHEK2, FBXW7,NOTCH1, RB1, CDKN2A, BAP1, FOXO3* and *MSH6*
Increased gene expression	PD-L1, OPN, ORAOV2 and FAP
Altered signaling pathways	FGF2-FGFR3, EGFR/AKT/S6 (EGF- EGFR), TGFβ- TGFβR3, Wnt pathway, PI3K pathway
Splicing isoforms	Cyclin D1 (Cyclin D1b), FIR (isoforms lacking exon 2), FGF (variant b), GHRHR (variant 1), NGAL (NGAL-2 and NGAL-3), LOXL2, MAGE-A10 (isoform with exons 3A and 3B), MUC1 (C, D and Z isoforms), PHF6(intron retaining isoforms), SRSF5, TCF4
**miRNA**	
Upregulated biomarker miRNAs	miR-1269, miR 142-3p, miR 181b, miR 223, miR 146b, miR 20b, miR 23, miR 129, miR 138, miR 151
Upregulated miRNAs that can be targeted by an inhibitor	miR 21, miR 25, miR 34b, miR 96, miR 128b, miR 130b, miR 330, miR 373
Downregulated biomarker miRNAs	miR 27b, miR 103, miR 34c, miR 140, miR 143, miR 145, miR 205, miR 518b, miR 30a-3p, miR 31, miR 92a, miR 106a, miR 133a, miR 133b, miR 148a, miR 150, Let-7d
Downregulated miRNAs that can be supplemented with mimics for treatment	miR 26a, miR 29c, miR 99a, miR 100, miR 107, miR 203, miR 296, miR 340, miR 375
Diagnosis and screening miRNA polymorphism in South African populations	miR 3184 (rs6505162) overlap of two oppositely orientated miRNAs, miR314 and miR413. (*NSRP1*)

## Data Availability

Not applicable.
